# Sequences and consequences

**DOI:** 10.1098/rstb.2009.0221

**Published:** 2010-01-12

**Authors:** Sydney Brenner

**Affiliations:** 1 King's College, Cambridge CB2 1ST, UK; 2 Crick-Jacobs Center, Salk Institute of Biological Studies, 10010 North Torrey Pines Road, La Jolla, 92037 CA, USA

**Keywords:** Cellmap, systems biology, biological information system

## Abstract

The conversion of data into knowledge constitutes a great challenge for future biological research. The new science of Systems Biology claims to be able to solve the problem but I contend that this approach will fail because deducing models of function from the behaviour of a complex system is an inverse problem that is impossible to solve. In addition, one cannot easily escape into high-level holistic approaches, since the essence of all biological systems is that they are encoded as molecular descriptions in their genes and since genes are molecules and exert their functions through other molecules, the molecular explanation must constitute the core of understanding biological systems. We then solve the forward problem of computing the behaviour of the system from its components and their interactions. I propose that the correct level of abstraction is the cell and provide an outline of Cellmap, a design for a system to organize biological information.

The double-helical structure of DNA was discovered by Watson and Crick in 1953 and in the remarkably short period of time of about a decade, molecular biologists had achieved a detailed understanding of the molecular mechanisms involved in gene replication and expression. The invention of two techniques in the mid-1970s, cloning and sequencing DNA, gave geneticists direct access to the sequences of genes. It led to the complete sequences of genomes, initially those of bacteria and yeast, then of *Caenorhabditis elegans* and *Drosophila*, two favoured model organisms, and finally, by the end of the century, less than 50 years after the initial discovery, to a complete sequence of the human genome. The last decade has seen many changes; sequence information is still growing exponentially and with the continued improvement and innovation in technology the pace of research has increased in all the life sciences. We now have unprecedented means of collecting data at the deepest molecular level of living systems and we have relatively cheap and accessible computer power to store and analyse this information. There is, however, a general sense that understanding all this information has lagged far behind its accumulation, and that the sheer quantity of new published material that can be accessed only by specialists in each field has produced a complete fragmentation of the science. No use will be served by regretting the passing of the golden years of molecular genetics when much was accomplished by combining thought with a few well-chosen experiments in simple virus and bacterial systems; nor is it useful to decry the present approach of ‘low input, high throughput, no output’ biology which dominates the pages of our relentlessly competing scientific journals. We should welcome with open arms everything that modern technology has to offer us but we must learn to use it in new ways. Biology urgently needs a theoretical basis to unify it and it is only theory that will allow us to convert data to knowledge.

Sequencing the human genome was once likened to sending a man to the moon. The comparison turns out to be literally correct because sending a man to the moon is easy; its getting him back that is difficult and expensive. Today the human genome sequence is, so to speak, stranded on a metaphorical moon and it is our task to bring it back to Earth and give it the life it deserves. Everybody understood that getting the sequence would be really easy, only a question of 3M Science—enough Money, Machines and Management. Interpreting the sequence to discover the functions of its coding and regulatory elements and understanding how these are integrated into the complex physiology of a human being was always seen as a difficult task, but since it is easier to go on collecting data the challenge has not really been seriously taken up. I am sure that there will be many readers who will deny this and claim that there already is a way of confronting this problem through a new branch of biological research called Systems Biology. This is precisely the main target of my article; I want to show that the claims of radical systems biology cannot, in reality, be met and that it will not be possible to generate unifying theories on that basis. There is a watered-down version of systems biology which, to my mind, does nothing more than give a new name to physiology, the study of function and the practice of which, in a modern experimental form, has been going on since at least the beginnings of the Royal Society in the seventeenth century.

It is not easy to find rigorous definitions of the aims and methodology of systems biology, but anti-reductionism is certainly one of its roots. The holistic approach is based on the idea that complex wholes cannot be understood by a study of the isolated parts. It is argued that when many components are put together, especially with interactions that are nonlinear, there are new emergent properties which can only be comprehended in the context of the whole system. Radical anti-reductionists contend that emergent properties are irreducible, and some believe that it is nonlinearity that distinguishes an emergent property, and that anything calculable by linear superposition of the properties of the parts is not emergent. In essence, the proposition is that molecules tell us nothing about cells and their behaviour, and neurons tell us nothing about brains and how they work. The second main root of systems biology is technological and comes from the application of methods of making many parallel measurements at the same time. My contemporaries will remember that this is not new. We screened large numbers of bacterial colonies by replica-plating and hand-arrayed phage plaques by pick and stab methods. We also had 96-well plates, but the application of miniaturization and techniques used in the computer chip industry has increased the scale of arrays from hundreds to hundreds of thousand features. It is also worthwhile considering why such techniques can be readily applied to the analysis of populations of nucleic acid molecules. To distinguish many different molecules we need an equal number of binding partners, each specific for one member of the population. The double-helical structure of DNA tells us that for any nucleic acid sequence we can immediately define a specific affinity reagent in the form of the inverse complementary sequence. Thus, we can generate large arrays of nucleic acid sequences by chemical synthesis or from double-stranded versions of the molecules themselves, which with reporters allow us to measure the abundances of a vast range of molecules. For other cellular components, such as proteins and carbohydrates, affinity reagents cannot be computed but have to be derived empirically in the form of antibodies or other molecules selected from large libraries which can be synthesized in parallel. Nonetheless, there has been a burgeoning of ‘omic’ sciences—proteomics, glycomics, metabolomics—echoing genomics, either enabling multiple parallel quantitative measurements of the components of cells or the systemization of existing data. Techniques have also been developed to measure protein–protein interactions either by expressing the proteins in yeast cells and coupling the binding to a transcription assay, or by using some display method to select binding partners *in vitro*. The analysis of micro-array data for gene expression is still considered a challenging problem for bioinformatics, but it is assumed that it will allow the identification of important cellular networks and thus lead to models of cellular behaviour. Indeed, there are some who think that all that will be required is the collection of more and more data under many different experimental conditions and then the right computer program will be found to tell us what is going on in cells.

I want to show here that this approach is bound to fail, because even though the proponents seem to be unconscious of it, this claim of systems biology is that it can solve the inverse problem of physiology by deriving models of how systems work from observations of their behaviour. It is known that inverse problems can only be solved under very specific conditions. A good example of an inverse problem is the derivation of the structure of a molecule from the X-ray diffraction pattern of a crystal. This cannot be achieved because information has been lost in making the measurements. What is measured is the intensity of the reflection, which is the square of the amplitude, and since the square of a negative number is the same as that of its positive counterpart, phase information has been lost. There are three ways to deal with this. The obvious way is to measure the phase; the question then becomes well-posed and can be answered. The other is to try all combinations of phases. There are 2^*n*^ possible combinations, where *n* is the number of reflections; this approach might be feasible where *n* is small but is not possible where *n* is in the hundreds or thousands, when we will exceed numbers like the total number of elementary particles in the Universe. The third method is to inject new *a priori* knowledge; this is what Watson and Crick did to find the right model. That a model is correct can be shown by solving the forward problem, that is, by calculating the diffraction pattern from the molecular structure. The universe of potential models for any complex system like the function of a cell has very large dimensions and, in the absence of any theory of the system, there is no guide to constrain the choice of model. In addition, most of the observations made by systems biologists are static snap-shots and their measurements are inaccurate; it will be impossible to generate non-trivial models of the dynamic processes within cells, especially as these occur over an enormous range of time scales—from milliseconds to years. Any nonlinearity in the system will guarantee that many models will become unstable and will not match the observations. Thus, as Tarantola (2006) has pointed out in a perceptive article on inverse problems in geology, which every systems biologist should read, the best that can be done is to invalidate models (in the Popperian sense) by the observations and not use the observations to deduce models since that cannot be successfully carried out.

Is there another way forward? There is and we can discover it easily from the consideration of some basic principles. First, I point out that, in a strong sense of the word, the whole living world operates as a reductionist system. This is because what is handed down from an organism to its progeny is not the organism itself but a description of it written in the molecular language of DNA of its genes. All the properties of the organism are ‘reduced’ to this molecular description. Genes are molecules and the genome is interpreted by translation into other molecules. How to understand the conversion of this molecular language into the organism is the central problem confronting biology. Wriggle though they may, systems biologists cannot escape the molecular level; it is of the essence of life. I once heard a Buddhist priest answer the question ‘What is the Bhuddist definition of Life?’ by saying ‘Many Bhuddists think that everything is alive: mountains are alive, rivers are alive’. I interrupted him saying ‘Mountains are not alive’ and when he asked me how I knew I replied ‘You can't clone a mountain’. Mountains do not contain internal representations; they are products of the laws of physics. What most people have forgotten in their easy dismissal of molecular biology is that it introduced the notion of information into biology and showed that it had a material basis in the form of nucleic acid sequences. It forces us to think of biological systems as molecular information processing systems rather than systems involved merely in the molecular processes of energy transactions and chemical transformations.

The genome must therefore form the kernel of any theory we construct but since transforming the information in a genome into the final living organism involves many complicated processes mediated by molecules specified in the genome, all of this will need to be known in considerable detail before we can read and understand genomes. There is no simple way to map organisms onto their genomes once they have reached a certain level of complexity. Thus while the genome sequence is central, it is a level of abstraction which is too cryptic to be used for the organization of data and the derivation of theoretical models. Proposals to base everything on the genome sequence by annotating it with additional data will only increase its opacity.

The correct level of abstraction is the cell. The cell is the fundamental unit of structure, function and organization of living systems—something we have known for 180 years. This is the key feature of what I have called Cellmap, a design for a biological information system that will allow us not only to handle the vast accumulation of data but also to generate and test hypotheses. Cellmap is at once a map of the molecules within cells and a map of the cells in the organism; for microbes the cell is also the organism. All of us started as a single cell that multiplied to produce more cells, which differentiated into many different cell types to make up the tissues and organs responsible for our physiological functions. In choosing the level of the cell we avoid the question of whether our analyses should be top-down or bottom-up; instead, our approach is middle-out, because from the vantage point of the cell we can look down on the molecules that constitute it and look up at the organism that contains it. Furthermore, we can adopt a uniform conceptual architecture for all levels, viewing the organism as a network of interacting cells in the same way as we view the cell as a network of interacting molecules. As we shall see later, cell functions are generated by specific agglomerations of molecules just as physiological functions are exerted by specific collections of cells constituting our organs. In this way, our approach directly reflects the structure of biological systems and, as we reduce each level to the level below—organisms to cells and cells to molecules—we can then confidently complete the reductionist programme because the properties of molecules can be reduced to physics. This cannot be done in one step; we cannot decompose a human being into elementary particles and ask for the probability that these reassemble into the same human being, with the same genes, immune system and memories. This is absurd reductionism and if it could happen it would indeed be a miracle. Humans are not made in nature by the condensation of particle gases; as, is well known, each arises as a zygote produced by the fusion of two kinds of germ cells from the two parents. Interposed between quantum mechanics and a living organism are multiple levels of organization controlled by genes which have been generated by the processes of evolution, each step producing changes in the genetic material followed by natural selection of successful phenotypes. I was once accused by Rene Thom of being a constructivist, which I understand was worse than being called an empiricist; I replied that I took pride in it.

In order to show how Cellmap can deal in detail with a complex system such as a cell, we look at some elementary features of cell structure and function. Any mammalian cell has about 20 000 active genes each producing a polypeptide chain, and we may ask how are we to understand the function of cells through these molecules and their interactions? It is unlikely that we can find a set of differential equations governing these activities and which might allow us to calculate the behaviour of the system. I have always found it advisable when confronted by such questions to analyse how the biological system itself has solved the problem. We first notice that single polypeptide chains hardly ever act alone, but are assembled with others into molecular devices that perform the function. Thus, splicing of DNA is carried out by an assemblage composed of the products of at least 65 genes; chain initiation in protein synthesis is carried out by a complex of factors with a total of 26 subunits. If we assume that the average number of components is 10, then such assemblages immediately provide an order of magnitude reduction in complexity and allow us to deal with about 2000 devices instead of 20 000 polypeptide chains. Furthermore, the cell is not a homogeneous solution of molecular entities but is divided into compartments: plasma membrane, lysosomes, Golgi apparatus, endoplasmic reticulum, mitochondria, nucleus and so on, and this provides another order of magnitude reduction in complexity. Thus, in each compartment, on average, we need to focus only on about 200 devices, the interactions among them and their communications with other compartments. Several features of this organization should be emphasized: firstly, we can make a distinction between strong interactions which govern the assembly of the devices and weak ones which are involved in the interactions between devices. The former are, of course, encoded in gene sequences that specify the amino acid sequences of interacting peptides as well as the entire tertiary structure which presents them in the right configuration. This is the way they are represented in the genome. The weak interactions often involve other molecules, ranging from small chemical messengers such as cyclic AMP to proteins that mediate the communication between devices and between compartments. The whole may therefore be pictured as a communication system, with devices transforming and passing information to each other. Even a biosynthetic enzyme pathway can be viewed in this way, with the substrate as the input message to an enzyme and the product, the output message, which itself may be an input message to another enzyme. This suggests that everything can be represented as a graph, with the devices at the vertices and their communicating messages as the arcs. The second important feature of this organization is that it illustrates an important property of biological systems that have evolved by piecemeal changes in the genome followed by natural selection. The modular structure makes this possible by confining the consequences of changes to a limited part of the structure without ramifying effects. But, more importantly, it reveals the great principle that biological systems solve many problems by treating them like income tax. As is well known, it is criminal to evade income tax, but there are perfectly legal means of avoidance. Thus in this case, the problem of molecular complexity has not been directly solved, but avoided by the modular structure, which in turn simplifies it and also facilitates evolutionary change.

This model of a cell also allows us to deal with questions of cell regulation. Today, if we are asked to predict the effect of a drug for a receptor on the heart, our response is to kill an animal and test the drug directly by demonstration. However, if we knew the graph of devices through which the drug exerted its effects (for example, the membrane receptor-G protein device that transforms the binding of the natural ligand into an internal signal resulting in the synthesis of cyclic AMP, which acts on another device to release calcium ions and which in turn leads to changes in the molecular complex causing contraction), we could calculate the effects by knowing what each device does and how many devices there are in the cell. What is more, once we have established and understood what each device does we can submerge the details of its structure and simply concern ourselves with its transfer function. Control engineering has produced several ‘device languages’ which we can use; we can represent the functions as electronic circuits or mathematical equations. We can include the time dimension in this representation in the form of rate constants but we will often find that these are not simple feed-forward pathways but will be stabilized by feedback loops, which can be incorporated in the same scheme. Many of the devices involved in signal transduction pathways employ mechanisms that involve covalent modifications of proteins by phosphorylation to induce conformational changes. Hardly anybody ever addresses the fact that after such changes the device must be restored to its ground state so that it can respond again. It follows then that all of these devices must show oscillations and the period of this oscillation becomes an important parameter. Naturally, such oscillations will be observed under normal conditions only if all the devices in a cell act synchronously, which shows that we will need to acquire special ways of observing the behaviour of single molecular devices in living cells. This is the real experimental challenge in the study of cell physiology.

Cellmap will also need to represent all of the different cell types of the body. We still do not know how many cell types there are, nor do we have a good definition of a cell type. Is a neuron that has learnt something a different cell type from its naive neighbour? We think of cell types as non-contingent, that is, not dependent on outside factors, but there are many cases of conversion of one cell type into another, and many instances in development where differentiation is under the control of contingent factors. Differentiation implies alterations in gene expression, mediated by transcription factors, and a cell type is specified by a set of transcription factors. In principle, specification of this set should allow us to compute the total pattern of gene expression in a given cell type and this might then provide a reduced description of a cell since the number of genes for transcription factors is of the order of 10 per cent of the total number. Thus, transcription factors and, in particular, assemblages of them are special devices which not only interact with each other but also with special DNA sequences in the promoters of genes. These devices may still depend on contingent inputs and their output is a decision to transcribe or silence a gene. Cellmap should have the capacity to show how these patterns change during development; we should also be able to see all the changes in the transcription factors and the resulting changes in gene expression as we go from stem cells to a Purkinje cell in the cerebellum or a dendritic cell in the skin. It is also worthwhile reminding biologists that all the proteins in our bodies are continuously turning over, and therefore must be constantly replaced. Thus we are not systems that are written only once, but transcription, like the Red Queen, must be constantly running to keep us going, maintaining the functions of all our cell types. Can we define this reduced description of an organism from which we can compute the molecular properties of all the cells that maintain the organism as a going concern? This is a key component of the reductionist approach because through it we avoid the systems biology programme of making extensive measurements of gene expression to be used in deducing the internal state. We also simplify how data might be handled. We do not need to store a total description of all gene expression for each cell type because we can compute it from the reduced description. Not only could we then understand how we differ from chimpanzees, our close cousins, but we could then carry out gedanken experiments, such as computing the organism heterozygous for the two genomes. Would language be dominant or would chimpanzees have language suppressor genes? Could we design genomes to make centaurs, a hexapod organism with two digestive systems, two hearts and two respiratory systems? How did they reproduce? The Greeks made centaurs by substituting the torso of a man for the neck of a horse, making them all male, but perhaps they all only looked male and there were two kinds of horse components, which house the reproductive organs.

The alternative approach to systems biology is to solve a set of forward problems. The whole may be greater than the sum of the parts studied in isolation but the very existence of biological organisms tells us that it cannot be greater than the sum of the parts and their interactions. Cellmap aims to define the parts and their interactions and provide ‘wiring diagrams’ as models of the system which we can use to compute outputs that can be compared with observations. We note here that when we come to simulate such systems, computation is carried out in the machine language of the system since all of the objects in Cellmap are the molecular entities themselves and not some description of them. As I have pointed out before, it is this that constitutes a true simulation of the system and not merely an imitation of its behaviour. Exactly the same approach would be applicable to the next level of organization. We can treat brains as networks of neurons defined with sufficient properties to permit the construction of functional wiring diagrams which we can use to compute outputs. We do not in this case need to keep in view all of the molecules in a nerve cell but only those that are relevant to the function of the network. We treat the problem of how a cellular device is fashioned by its constituent molecules as separate from how these cellular devices function as units in neural networks. The framework of Cellmap places strong bounds on how much information we need to use to generate computable models of biological processes. It will enable us to trim and organize the vast amount of data that exists in the scientific literature to the essential measurements that are needed for the computational model. Similar ideas have been recently discussed by Nurse (2008), but we differ in that I do not think that we need to view the modules as ‘logic’ modules nor do we need new languages to understand the ‘management of information flow between logic modules’. I believe everything is there in the ‘hardware’ of the cell; there is no explicit ‘software’ level, except one in our minds. If we do need a new language to help us understand these processes I believe it should be a picture language that can be generated from the information in Cellmap and not more text. There is already too much text in the world. One good way to view the problem is as a new kind of molecular biology, the molecular biology of organization.

The basic fact that living systems are the products of evolution raises additional questions. We might like to think that organisms can be represented by a set of numbers which we should try to determine as accurately as possible. Such numbers as the affinity constants of enzymes or the number of molecules of a protein expressed in a cell will be relevant for Cellmap or for any other system. In fact, one of the criticisms of systems biology is that their measurements of the behaviour of systems are not accurate enough and noise emanating from many sources will blur the true values. We must recognize that these numbers are ultimately specified in DNA sequences in the genome: affinity constants depend on the peptide sequences lining the active sites of enzymes, and turning a gene on or off in a particular cell requires the appropriate recognition sequences for transcription factors. There is an evolutionary cost in fixing such numbers and changes will be selected if they have an effect on reproductive success. This means that there could be a third value—indifferent—in addition to good and bad, and these ‘don't care’ values immensely complicate the inverse approach. In the forward approach the values are determined as elementary properties of the molecules and are directly applied to the computation of the behaviour of the cell or organism. We need to remember that whereas mathematics is the art of the perfect and physics the art of the optimal, biology, because of evolution, is only the art of the satisfactory.

Finally, we can now see how Cellmap can be connected with the genome. In order to do this rigorously, we need a more precise definition of the gene, which we have been using very loosely as a DNA sequence specifying a single, unitary function. It is this loose definition of the gene that led to surprise at the finding that the human genome contains about 27 000 genes, which is only about six times the number found in the bacterium *Escherichia coli*. Actually, when genome sequences are analysed we find that many of the genes are used in multiply different ways. Often a gene may have two or more different promoters corresponding to the different cells in which it may be expressed as well as different protein products produced by alternative splicing with peptides added or removed. In many cases, these peptide sequences specify addresses within the cell, or confer specific functional properties. Thus, as pointed out before (Brenner 2000), it is better to talk about loci that occupy defined positions on the genome rather than genes and then specify what we may term the different instantiations of the locus. The significant values derive both from the number of loci and the number of different instantiations of each locus. Since most of the loci in micro-organisms have only one instantiation, the disparity between ourselves and *E. coli* will become more respectable. It is very likely that the compound structure of a locus is not only the consequence of evolution, but also facilitated it by allowing accretion not only to genomes as a whole but also to individual loci.

We may compare Cellmap with how our understanding of a city would be embodied in an analogous CITYMAP. The white pages of the telephone directory are like the genome sequence. We trust it to be accurate and complete and although it lists the people who compose the city it tells us little about how it might work. The yellow pages are comparable to the annotated sequence; they tell us a little more about function. Thus, a list of plumbers allows us to deduce that there will be pipes somewhere in the city because plumbers plumb pipes. However, the essential feature of the city is grasped only when we realize that there are units called homes in which families live; that in the morning, the families disassemble and the components then travel and aggregate with components from other homes in units such as schools, shops, banks, factories and that these are the functional units of the city. CITYMAP would need to embed knowledge both of the structure of these units and the flows between them. It would also find that the city had compartments, the city centre being distinct from the residential areas and that cities could differ widely as a consequence of their locations and history.

This article has been written to mark the 350th anniversary of the Royal Society in 2010 and expresses a personal perspective as requested by the Editor. It may be seen as an expansion of some earlier ideas (Brenner 1999). I hope I have shown that the programme of systems biology cannot be achieved because the inverse problem cannot be solved. The way forward is to continue in the path of molecular biology, unveiling how the genome expresses its information through proteins and other molecules, how these build assemblages and how the functions of these are integrated in the cell. Cellmap expresses a theory of how this integration is achieved; it also provides a database which, when complete, will allow us to formulate and test hypotheses by computation. This should become a major programme of biological research; it will not be done by one person nor even by one laboratory but will require the participation of the entire community of biologists. Which genome should we choose? Many will argue that this is best done for the genome of a moderately complex model organism such as *Drosophila* or *C. elegans* where a wide range of experiments, including genetic intervention, are possible. However, I believe that we should do it for the human genome because we need to know everything about our own biology to explain and understand our specific human capabilities. We generated an international programme for sequencing the human genome; we now need one for reading and interpreting it. Perhaps, on the 400th anniversary of the Royal Society our successors will be able to celebrate its conclusion.
